# Qualitative study of patients experiences and perceptions of stepping down asthma medication in primary care across England

**DOI:** 10.1136/bmjresp-2024-002898

**Published:** 2025-02-03

**Authors:** Chloe I Bloom, Jack Middleton, Adam Lewis

**Affiliations:** 1 National Heart and Lung Institute, Imperial College London, London, UK; 2 Oxford University Hospitals NHS Foundation Trust, Oxford, UK; 3 School of Health Sciences, University of Southampton, London, Middlesex, UK; 4 Brunel University, Brunel University London, London, UK

**Keywords:** Asthma, Asthma Epidemiology

## Abstract

**Background:**

Guidelines recommend that asthma medication should be stepped down to the minimally effective dose that achieves symptom control. Stepping down aims to prevent adverse medication effects and unnecessary costs but is often not implemented in primary care, where most patients with asthma are managed. Little is known about the experiences and views of patients regarding stepping down.

**Methods:**

Patients with stable asthma, with regular use of a preventer inhaler, from general practitioner practices across England, participated in a survey and/or semi-structured interview. Questions explored the patient’s understanding of their asthma, views and knowledge of preventer inhalers, experiences and perceptions of stepping down asthma medication. Qualitative group-based multidisciplinary thematic analysis by two healthcare professionals and a patient were performed.

**Results:**

143 patients responded to the survey, 63% were female, between the ages 18–80 years and including geographical areas across the UK, 17 of whom participated in an interview. Half of these patients with stable asthma, most with asthma for more than 10 years, had never had a discussion regarding stepping down asthma medication. Most stepping down that had occurred was related to seasonal changes in asthma control. Four overarching themes from the interviews were identified, (1) experiences of living with asthma and needing inhalers, (2) insufficient education regarding preventer inhalers, (3) stepping down is agreeable and possible and (4) current asthma care is suboptimal.

**Conclusion:**

Patients with stable asthma were able to self-manage their asthma well. They had little awareness of medication adverse effects and minimal experience of having their medication stepped down by a healthcare professional. Most were inclined to step down, if clinically safe to do so, indeed some had reduced their medication doses themselves, without professional guidance.

**WHAT IS ALREADY KNOWN ON THIS TOPIC**

**WHAT THIS STUDY ADDS**

- Patients are amenable and keen to step down their asthma medication doses, when clinically appropriate.

**HOW THIS STUDY MIGHT AFFECT RESEARCH, PRACTICE OR POLICY**

- Patient willingness should not be a barrier to stepping down; primary care clinical trials and clear guidelines on how to step down are needed to prevent the overuse of asthma medication.

## Introduction

 Approximately 1 in 12 people in the UK have asthma that requires regular inhaled medication.^1^ Over 95% of those with asthma are managed solely within primary care, often by healthcare professional (HCP) without specialist asthma knowledge.^2^ A key aspect of asthma care is the pharmacological management of worsening symptoms and asthma attacks. Asthma guidelines recommend a stepwise approach to increasing medication and provide clear guidance on how to ‘step-up’.^3^,^5^ Guidelines also recommend patients are only prescribed the maximal dose of inhaled medication required to obtain optimal asthma control. Unfortunately, the advice on how to step-down medication is much less formalised. This lack of clarity has been found to be one of the key reasons why primary care professionals are familiar with stepping-up, but rarely step-down medication.^6^ This has also likely contributed to the increasing prescriptions of higher doses of preventer medication over the past two decades, leading to unwarranted higher doses of corticosteroids.^7^

There are numerous disadvantages related to the overtreatment of inhaled corticosteroids; consequences include the increased risk of side effects, such as cardiovascular disease, pneumonia, pulmonary embolisms, cataracts, osteoporosis and diabetes.^8 9^ Another concern is the large cost burden to the National Health Service (NHS) as inhalers contribute to around 13% of the primary care prescribing budget.^10^ Excess inhaler use also results in a higher carbon footprint.^11^ Lastly, overtreatment causes unnecessary medication burden for patients, which may contribute towards poor adherence.^12^

We have previously attempted to understand the barriers to stepping down asthma medication from the viewpoint of primary care HCPs, including general practitioners (GPs), nurses and community pharmacists from across the UK.^6^ We conducted a mixed methods qualitative study and found four key themes, low awareness of the need to step down, inertia to step down, poor self-efficacy for the ability to step down and lack of feasibility.

But to fully appreciate how to overcome the barriers to stepping down, we sought to understand the perceptions and experiences of patients. Here, we have conducted a mixed methods study, approaching patients who have stable asthma managed solely by their GP practice.

## Methods

### Study design

The study adopted a qualitative methodology, using a questionnaire and semi-structured interviews from a convenience sample of primary care, stable, patients with asthma.

### Patient and public involvement

Eight patients were involved in the design of the study, input into the participant information sheets (PIS), formation of questionnaires, interview questions, coding of the interview scripts (JM), reporting of the findings (JM) and reviewing of the manuscript draft. None of the patient and public involvement (PPI) members were a participant in the study.

### Patient recruitment

Recruitment was facilitated through the Clinical Practice Research Datalink (CPRD). CPRD is a real-world research service that covers a network of UK primary care practices, its research services are delivered by the Medicines and Healthcare Products Regulatory Agency with support from the National Institute for Health and Care Research.

Patients were eligible if aged 18–80 years, attended a CPRD-linked GP practice and have stable asthma (≥4 preventer inhalers, <3 reliever inhalers and no asthma attack in the previous 12 months). Invites were sent out by 41 CPRD-linked GP practices that were Research Ready as per the Royal College of General Practitioners.

Eligible patients received an email, or letter, where they were invited to take part in a questionnaire. If they volunteered, they were sent a personal code to access the Research Electronic Data Capture (RED Cap) database and PIS. At the end of the questionnaire, they were invited to participate in an interview. The researcher contacted consenting volunteers to participate in an interview using purposive sampling, based on sex, age and geographical location.

All participants provided informed consent, including the publication of their anonymised responses; participants were told the length of time of the survey and interview, where the data was stored and for how long, who the investigator was and the purpose of the study. Survey and interviews were completed between February 2023 and January 2024.

### Data collection

#### Face validity

Potential questions were discussed with three PPI members before drawing up the questionnaire and interview question guide.

#### Content validity

The questionnaire and interview guide were piloted, developed, modified and informed by five different PPI members, as well as a review of the literature.

We drew up a 13-question survey intended to assess participants knowledge about the medication in their preventer inhaler and any experience of stepping down (online supplemental table 1). Participants were able to review and change their answers. Duplicated entries were avoided as the participant-individual survey code could only be applied once. Incomplete questionnaires were not accepted by REDCap.

The interview question guide was designed to explore views and experiences around stepping down and consisted of open-ended questions, that were semi-structured and around the following topics (online supplemental table 2):

- Understanding of their asthma.
- Views on preventer inhalers.
- Knowledge on preventer inhalers.
- Experiences of stepping down asthma medication.
- Views on stepping down asthma medication.

One-to-one interviews were carried out by CIB (female researcher at Imperial College London and honorary respiratory consultant at Imperial College Healthcare NHS Trust) remotely through video or telephone with participants in their homes. All interviews were digitally recorded and transcribed verbatim.

### Data analysis

All completed surveys were analysed. Survey responses and demographic information from the interviews were summarised using frequencies and percentages.

Interviews were analysed according to the principles of both group-based multidisciplinary qualitative work and reflexive thematic analysis.^13 14^ These methodological decisions were made based on a framework of knowledge gained from the author’s previous professional interviews focused on stepping down inhaled medications and survey data presented here, which enabled, to some extent, deductive analysis, as this were based on the previous knowledge. These methods also accounted for multiple HCPs being involved in the analysis (physiotherapist and doctor), while at the same time a person living with asthma and a prescriber, valuing the importance of reflexive inductive analysis in the process. Analysis was facilitated using NVivo (Lumivero). Following transcription both JM and CIB independently coded the data and developed provisional themes. An example of a reflexive report made during this stage can be found in the online supplemental data. JM, CIB and AL met repeatedly to discuss codes and provisional themes, before further analysis reviewing the transcripts with working themes, re-working and finalising them. Interpretation of the data is provided in the results and discussion sections below.

The Checklist for Reporting Results of Internet E-Surveys (online supplemental file 1) and Consolidated criteria for Reporting Qualitative research (online supplemental file 2) were used to guide reporting.Ethics

## Results

### Questionnaire: participants

143 participated in the questionnaire, 90 (63%) were female and the proportion by age were, by years: 18–29=4%, 30–49=23%, 50–69=50%, 70–80=23%. Participants were registered with 15 different GP practices, from the following regions, Northwest Coast, West Midlands, West of England, Wessex, North Thames, Yorkshire and Humber, Thames Valley and South Midlands.

### Questionnaire: frequency of discussing stepping down

94% use their preventer inhaler most days. One-third were unaware, or unsure, if their inhaler contained corticosteroids (table 1). 54% had never discussed stepping down. One-fifths had discussed stepping down, more than once, with an HCP. One-quarter had intentionally stepped down their preventer themself.

**Table 1:** Questionnaire responses

Questions	Response	Total (%)
**Do you use your preventer most days**	Yes	135 (94.4)
**Are you aware your preventer inhaler contains steroids**	YesNoNot sure	97 (67.8)5 (3.5)41 (28.7)
**Has anyone ever discussed reducing your inhaled medication with you?**	Never	89 (62.2)
	Once	26 (18.2)
	Several occasions	28 (19.6)
**Have you ever asked if you can reduce your inhaled medication?**	Never	108 (75.5)
	Once	20 (14.0)
	Several occasions	15 (10.5)
**Have you ever purposely used your preventer less often?**	Yes	35 (24.5)
	No	107 (74.8)
	Not sure	1 (0.7)
**If have purposely used preventer less often, it is because one of more of the following:**		
My asthma is better at certain times of the year		26 (74.3)
I do not like taking medication		8 (22.8)
Reduce risk side effects		8 (22.8)
Reduce my costs		1 (2.8)
Other		4 (11.4)
**Would you reduce your inhaled medication if a healthcare professional suggested to:**		
Yes - I prefer to use less medicine		104 (72.7)
Yes - I worry about side effects		17 (11.9)
Yes - as it saves me money		5 (3.5)
No - I would still worry asthma may worsen		33 (23.1)
Other		9 (6.3)
**Would you be happy to reduce your inhaled medication by using a different inhaler**	Yes (if shown how)	112 (78.3)
	No	12 (8.4)
	Other	19 (13.3)
**Have you ever had side effect(s) from your inhaled medication?**	Yes	27 (18.9)
	No	102 (71.3)
	Not sure	14 (9.8)
**If you have experience symptoms, what are they?**	Mouth/throat related	14 (59.2)
	Excess bruising	3 (11.1)
	Palpitations	3 (11.1)
	Shaky/tremor	3 (11.1)
	Not reported	4 (14.8)

### Questionnaire: beliefs around stepping down

From those that had stepped down, three-quarters did so because of seasonal changes in their asthma symptoms. Only 23% stepped down because of side effect concerns. Many were unconcerned about adverse medication effects, for example, in the free text box:

I am happy with my current inhaler and see no reason to change it*.*

I am not aware of any side effects and always make sure to clean my teeth afterwards as advised by my dentist.

One patient noted costs as a reason they had stepped down.

When asked if they would reduce their asthma medication, if an HCP suggested to, three-quarters, agreed they would. Around 10% said they would agree due to concern about side effects. 5% would step down because of financial benefits.

A quarter of patients, even if advised by the HCP, would not agree to step down, related to worry of loss of their current good asthma control.

Maybe but I don’t want my asthma to get worse. Using the steroid inhaler has controlled my asthma much better than anything I have used before.

I do not want to change inhalers as the one I have been using for several years now is working very well and my asthma is under control.

78% would be happy to step down by switching to a completely different inhaler that they were shown how to use.

### Questionnaire: medication side effects

20% had experienced side effects from their preventer, including local effects in their mouth or throat. A small number reported systemic symptoms including excess bruising, palpitations and hand tremors.

### Interviews: study participants

17 patients consented to an interview. 11 women and of the following ages, by years: 18–29=1, 30–49=4, 50–69=9 70–80=3; residing in eight areas across the UK: Cornwall, Gloucestershire, Herefordshire, Cumbria, Birmingham, Hampshire, Norfolk and Blackpool. Interviews lasted 25–40 min.

### Interviews: overarching themes

Four common themes were identified, (1) experiences of living with asthma and needing inhalers, (2) insufficient education regarding preventer inhalers, (3) stepping down is agreeable and possible and (4) current asthma care is suboptimal (table 2). Each theme is discussed below, supported by verbatim quotes.

**Table 2:** Example codes and quotes aligned to themes

Major themes	Quotes	Codes
**Experiences of living with stable asthma**	‘I’ve had it for so long. It’s just part of me.’ (Participant 9)‘I’m so really, just used to it now, to be quite honest, it’s part of my morning routine*.*’ *(*Participant 11)‘I have just kind of got used to managing it myself.’ (Participant 15)‘I try not to worry too much. I’d probably just think, well, if the same happens as last time, then I know what to do*.*’ (Participant 17)	Accepting of inhalersAsthma under controlForget I have asthmaAsthma all my lifeHad bad asthmaInhalers are necessaryLong-term asthmaInhalers keep me aliveNot like asthmaRoutine for asthmaSelf-manageAsthma control
**Insufficient education regarding preventer inhalers**	‘….so they can bulk you up as far as I understand it.’ (Participant 1)‘No I haven’t [read anything about inhalers and the environment). But is that why I’ve got this white one?’ (Participant 8)‘No one’s ever mentioned it to me, but I can certainly imagine that they are.’ (Participant 15)‘No, only the blue one.’ (Participant 11)	Change puffs when worse symptomsChange inhaler dose or typeCombination inhalersConcern about side effectsLack of awareness of side effectsInaccurate knowledge on side effectsEnvironment knowledge of inhalersNot know had steroids in inhalersNot question the doctorPoor communicationRight inhaler is game changerSelf-managementSpacerPeak flow monitoring
**Stepping down is agreeable and possible**	‘But I’m on one a day, one morning and one evening. I do wonder if I could reduce that*.*’ (Participant 5)‘I prefer to use mine less often if I could…. But they are a right old drag to be fair, and you got to keep ordering them, and you had to pay for it before I was 60.’ (Participant 8)‘I may be something that’d be willing to try……. Then, if I can take less of it, it means it lasts longer.’ (Participant 10)‘I’d be willing to give anything a go. To be honest I do find, even though, like I said, they are a crutch for me, but also that I would love to not even be on them.’ (Participant 11)‘That wouldn’t bother me. I think it would be a good thing. An annual review would probably be the best time to do it.’ (Participant 9)‘I think if they were planning on doing it, I think it would best on an annual review to speak face to face about it….’ (Participant 16)‘And if it was a sensible, viable alternative, and I didn’t discover that they were just doing it on a cost basis, then it you know, I would consider it…’ (Participant 3)	Change puffs on ownReduced it with weather changesChange in inhaler dose or typeAlter puffs due to symptomsGP switch my inhalerNo one told me I could reduce itTold me I could not reduce itPrefer not to use inhalers at allReassurance if changeDose reduced by trial and errorExperience of dose reduction
**Current asthma care is suboptimal**	‘Just really a telephone call. They just say, how is it being? And you know, what is your peak flow and weight…. I’m guessing but what I think is happening is it’s box ticking.’ (Participant 6)‘Yeah, I think the paper may have been there 4, or 5, years ago.’ (Participant 1)‘No not really, I’ve got a peak flow.’ (Participant 4)‘Oh, I think that will be marvellous. That would really appeal to me….on a longer-term thing, well, yes, because you’d know what you could do then…. And its just that bit of reassurance to look at…’ (Participant 17)‘Well, when I first went on them, I said, how long with these be for? And I said, is it gonna be for life? She said, well, if you have got asthma, you are on them forever.’ (Participant 8)	Check-upsBlanket approach to switching everyoneProfessionals giving different informationNot receiving education or informationBrief phone conversationText messagesNot seen a doctor for yearsPrefer to see someone in personSelf-manage wellSymptoms not peak flow are useful

GPgeneral practitioner

### Experiences of living with asthma and needing inhalers

All but one participant had asthma diagnosed over 10 years ago. Many spoke of their acceptance and understanding it was a lifelong condition, only a few had ever asked an HCP if they might achieve remission.

I don’t really think its gonna go away. So I’ve kind or resigned to the fact that I’ve got asthma. (Participant 12)

I just assumed is for life cause its not got any better. (Participant 13)

There was general acceptance of needing to use their asthma inhalers every day.

*…*its just a part of my routine, like it’s the last thing I do before I go to bed, like when I’m putting my moisturiser on and take my make up off and stuff*…* (Participant 10)

I guess I’m happy with inhalers, because they’ve just over the last years become a way of life*.* (Participant 17)

Participants felt they were aware of their asthma deteriorating by their symptoms, without the use of peak flow monitoring and most people self-managed their asthma.

I do it on symptoms not peak flow. I find a peak flow is too easy to manipulate*.* (Participant 3)

I go on, how I feel because I’ve had this so long*.* (Participant 7)

Furthermore, most felt they self-managed their asthma.

*…*cause I’ve had it so long and because I don’t go to the doctors very often, I don’t see anybody, so I do feel like I am just managing it myself. (Participant 11)

### Insufficient education regarding preventer inhalers

Some participants did not know that their preventer inhaler contained steroids. One patient reported that their doctor did not know. Even those who did know, often were not aware of what the possible side effects were, or incorrectly thought the steroids were anabolic steroids.

I went into the doctors and I said…. ‘of course, my inhalers have got steroids in. And he says ‘No they haven’t got any in at all*.*’ (Participant 8)

I know my skin appears thin, I don’t know if that’s a steroid thing? (Participant 13)

No, its never really brought to my attention. (Participant 15)

Some participants feel they manage their asthma themselves, because they have not been told how to by a professional.

Nobody has told me. I just used both my inhalers more. I’m not sure if I am doing the right thing but my chest was tightening*.* (Participant 4)

If I’m a bit wheezy, say at night, and I take the salbutamol first before taking the Fostair as I think it opens up the tubes a bit*.* (Participant 2)

Most participants were not aware of links between the environment and inhalers.

No I haven’t [read anything about inhalers and the environment]. But is that why I’ve got this white one? (Participant 8)

No one’s ever mentioned it to me, but I can certainly imagine that they are*.* (Participant 15)

On asking if an HCP had ever discussed stepping down their preventer medication, many had no experience of that.

No, its not been a discussion with asthma but it has with my blood pressure tablets*.* (Participant 13)

No, I haven’t been told to change it. But then I don’t have much contact with the respiratory nurses. Sort of a yearly review, unless there’s something wrong*.* (Participant 17)

### Stepping down is agreeable and possible

Several patients had previously stepped down their medication themselves and others had thought about it, but not yet tried it, often related to seasonal asthma changes.

I think I’m taking the limit on Fostair, and I think perhaps certain times of the year I actually don’t need to take that much*.* (Participant 2)

But I’m on one a day, one morning and one evening. I do wonder if I could reduce that*.* (Participant 5)

I think in my head a bit in the summer, and I think, you know, even though I know you’ve got to keep taking it but can I cut back? (Participant 7)

When asked if they would be happy to step down their asthma medication, if an HCP advised it, most were amenable to this. Reasons included reducing their effort to obtain their inhalers, reducing costs, preference not to take corticosteroids.

I don’t really want, you know, take any of the medication at all*.* (Participant 6)

I prefer to use mine less often if I could…. But they are a right old drag to be fair, and you got to keep ordering them, and you to pay for it before I was 60*.* (Participant 8)

I may be something that’d be willing to try……. Then, if I can take less of it, it means it lasts longer*.* (Participant 10)

I’d be willing to give anything a go. To be honest I do find, even though, like I said, they are a crutch for me, but also that I would love to not even be on them*.* (Participant 11)

Well I would rather not use inhalers. I’m aware they are steroids or something really don’t want to take but they control it*…* (Participant 13)

Two patients were not keen on trying to step down their asthma medication.

I don’t know, because it’s a case of if it works, you know its not broke don’t fix it*.* (Participant 7)

It has been discussed, and I’m usually reluctant to change things too much………. If its not broke, then lets not change it*.*(Participant 14)

On discussion about stepping down as a blanket approach for all patients with asthma at the GP practice (to help with NHS costs or the environment), most people were agreeable.

If they want to save money or the environment, as long as it it’s a similar product that will help me, its worth a try*.* (Participant 2)

I say, well, if that’s going to do the job that’s fine, I will have a go*.* (Participant 6)

If I knew it was going to do the same job with no additional side effects. I’ve not got an issue with that. I mean, you know I don’t pay and I know everything adds up*.* (Participant 13)

When asking how they would prefer blanket switching to be done, participants generally preferred it to be in person.

That wouldn’t bother me. I think it would be a good thing. An annual review would probably be the best time to do it*.* (Participant 9)

I think if they were planning on doing it, I think it would best on an annual review to speak face to face about it*….* (Participant 16)

The approach to stepping down was not a major concern although some did express a preference.

Well, I guess I would prefer the lower dose in the same inhaler so I could double the dosage again*.* (Participant 2)

*…*it wouldn’t bother me. I’ve had all different shapes*.* (Participant 16)

Other patients felt a blanket approach was not appropriate.

I think I would say you are only really able to do it on a patient-by-patient basis…some people could, you know be seriously harmed if it was a blanket approach*.* (Participant 7)

And if it was a sensible, viable alternative, and I didn’t discover that they were just doing it on a cost basis, then it you know, I would consider it*…….* (Participant 3)

### Current asthma care is suboptimal

Several patients felt asthma reviews were infrequent and a ‘box ticking’ exercise. Some people had their inhaler technique checked at their asthma review, but several did not, and many did not have an asthma management plan, or the plan was old.

Well, this [asthma review] on Friday is the first one I’ve had in probably 15 years*.* (Participant 2)

Yes, certainly before Covid…. and they always went through your technique. The last 2 or 3 years it’s been by phone*.* (Participant 13)

I take mine through a spacer, anyway, so no one checks that, but I haven’t had a review in person for 6 or 7 years*.* (Participant 15)

I know what you mean, and I have got the [asthma management plan] leaflet and I have filled it in, but it’s a bit of a noddy’s guide really*.* (Participant 14)

But those who had an asthma management plan still often used it.

The plan I’ve got now is all about day-to-day management, and its very good*.* (Participant 12)

As the asthma management plan is concerned with short-term management, they were asked if they would like something written that considered longer-term management.

I would say anything that can give you an idea of a future plan about either your own personal well-being, or possibly about your medication, in terms of how you or it might change over time would be enormously useful*.* (Participant 12)

Oh, I think that will be marvellous. That would really appeal to me. If I had something……. on a longer-term thing…because you’d know what you could do then…. And its just that bit of reassurance*….* (Participant 17)

Although it would not be suitable for everyone.

I probably look at it once and put it in the drawer, which is rather what I’ve done with the asthma management plan, because well, that was a bit of a wasted piece of paper, really*.* (Participant 14)

Participants rarely recalled discussing long-term and future planning with an HCP.

It’s very much about what’s happening today, so to speak, what’s the peak flow saying? (Participant 1)

They just ask how I manage it now, how I’m taking my inhalers*.* (Participant 11)

## Discussion

We explored the experiences and perspectives of patients regarding stepping down asthma medication. Most patients were able to self-manage their asthma and had been diagnosed years ago, thus were accepting of their condition and viewed it as part of their life, with medication use being a routine daily activity. In general, they were not aware of the potential adverse effects of their inhaled medication. Participants had minimal experience of having their medication stepped down by an HCP. However, the majority were willing to step down, if clinically safe to do so, and indeed some had reduced their medication doses themselves.

Many participants had asthma since childhood and had reached stability with their symptoms and inhaler use, which may explain why there was little impetus for the patients to suggest stepping down to HCPs and vice versa. There was often a lack of awareness and education on potential side effects from corticosteroids, either from inhaled or oral corticosteroids. The majority of side effects reported were local effects, not the more serious systemic effects that can occur, although some mentioned experiencing palpitations which could be cardiac in origin.^8 9^ Other potential drivers to stepping down, money and aggravation of a daily medication, were contemplated. Monetary considerations were a priority for very few but three-quarters of those surveyed would prefer to use less medication.

In general, patients had little education or discussion regarding long-term expectations of their asthma. For many, the understanding was that asthma would be a lifelong condition, but this was based on their own experiences. Without such discussions and a continued focus on acute, short-term goals, this limits the capacity to consider reaching a longer-term goal of minimal asthma medication use. As determined in our previous study of HCPs, there are multiple barriers to having such a discussion in a primary care setting.^6^ One of these aspects was a lack of feasibility, including time limitations, which was a common theme for patients who often felt their routine asthma clinical care was suboptimal and ‘box ticking’.

An area of discussion was the switching of inhalers at the GP practice level, occurring as a blanket switch of certain inhalers for all patients. This is a common approach across the UK yet is heavily criticised by the Primary Care Respiratory Society UK,^15^ although it has been found to have positive effects.^7^ Perhaps surprisingly, the concept of blanket switching was often well-received and understood, regardless of if the initiative was driven by NHS costs or potential environmental impacts. But equally some participants were opposed to the broad stroke activity, lack of personalisation being a critical reason.

There have been no previous studies assessing patients views and experiences of stepping down asthma medication as most studies regarding deprescribing have focused on elderly patients and the views of HCPs rather than the patients.^16 17^ Overall, the implication of this study is that patients are willing to step-down asthma medication and are less concerned about the worsening of their asthma control than HCPs believe. Here, we provide some evidence that we should be designing patient-centred trials to understand how best to safely step-down stable patients.

### Strengths and limitations

To our knowledge, this is the first study to explore the experiences and perceptions of patients regarding stepping down. Our participants represented a range of demography and geographical locations. To obtain a broader representation and a larger sample size we used both a questionnaire approach and interviews, the results of which are supportive of each other. We also provide repeated negative case analyses from the interviews, illustrated the complexity of asthma medication management from patient perspectives. There was an intentional selection bias in this study; we only included those with stable asthma, the only patients that stepping down is recommended for.

There are also several limitations. As we did not include patients with asthma that was not well controlled, we do not know their opinions. In the survey, we did not know if patient’s asthma was well controlled other than the inclusion and exclusion criteria as above. We only included adults and did not include adolescents or families of children with asthma. There were also fewer younger patients, 18–29 years, in the study and their views and experiences may differ. We only included people living in England, where views, particularly around prescription costs may have differed in other countries within the UK where all prescriptions are free. We did not know the socioeconomic status, comorbidities, smoking history or other factors that may have influenced the views and experiences of the participants. Our study only includes the views of those patients willing to complete the survey which may bias towards those more interested in their asthma treatment as the study invitation letter included ‘We are asking you because we want to hear patient’s views and experiences on their asthma inhalers. Part of the survey will ask if you or a healthcare worker has ever reduced your inhaler doses’. This may have led to a higher proportion of those surveyed and interviewed being open to the concept of stepping down their medication, although it is notable that the majority of them had not inquired about stepping down.

## Conclusion

This study found that patients had little experience of stepping down their medication by an HCP. In general, they felt they managed their asthma themselves and often experienced suboptimal asthma care. Many patients were willing to try stepping down if recommended by an HCP, yet most were not presented with that opportunity, potentially putting them at unnecessary risk of adverse effects. Future directions should include clear clinical guidance on how to safely step-down asthma medication, derived from pragmatic clinical trials conducted within primary care, alongside education of HCPs and patients, including on the longer-term prospects and management of asthma.

## supplementary material

10.1136/bmjresp-2024-002898 online supplemental table 1

10.1136/bmjresp-2024-002898 online supplemental file 1

10.1136/bmjresp-2024-002898 online supplemental file 2

## Data Availability

Data are available upon reasonable request. All data relevant to the study are included in the article or uploaded as supplementary information.
