# Supplementary material for: Qualitative study of patients experiences and perceptions of stepping down asthma medication in primary care across England
Source: BMJ Open Respir Res. 2025 Feb 3;12(1):e002898. doi: 10.1136/bmjresp-2024-002898 (PMC11795401; doi:10.1136/bmjresp-2024-002898)
Supplement: online supplemental table 1 [file bmjresp-12-1-s001.pdf]

Supplementary table 1. Questionnaire sent to participants

	Questions	Answer options
1	Do you feel your asthma has been mostly stable* for the past year?<br>*no asthma attacks in past year and minimal use of your reliever inhaler each week	Yes<br>No<br>Not sure
2	Have you used the same preventer inhaler for the past year or more?	Yes<br>No<br>Not sure
3	Do you use it most days?	Yes<br>No<br>Not sure
4	Does the preventer that you use contain steroids?	Yes<br>No<br>Not sure
5	Are there periods of time when you purposely try to use your preventer less often?	Yes<br>No<br>Not sure
6	If yes, why do you use it less (tick all that apply)	My asthma is better at certain times of the year<br>Because I don't like taking medication<br>To reduce the risk of side effects<br>To reduce costs<br>Other - please explain below
7	Has a healthcare professional discussed reducing how much you use your preventer?	Never<br>Yes - once<br>Yes - on several occasions
8	Have you ever asked a healthcare professional if you can reduce how much you use your preventer inhaler?	Never<br>Yes - once<br>Yes - on several occasions
9	Have you ever had side-effects from your asthma inhalers?	Yes, if yes please describe below<br>No<br>Not sure
10	If a healthcare professional told you, it was safe to reduce your asthma inhaler medication, would you try reducing it? (tick all that apply)	Yes - I prefer to use less medicine<br>Yes - I am worried about side effects<br>Yes - If it would save me money<br>No - I would be worried my asthma may worsen<br>Other - please explain below
11	If they suggested reducing the dose by using a different inhaler, would you agree? (tick all that apply)	Yes - I would be happy to if I was shown how to use it<br>No - I would be worried a new inhaler might be harder to use<br>Other - please explain below
12	What is your gender?	Male<br>Female<br>Other
13	What is your age group?	18-29<br>30-49<br>50-69<br>70-80

Supplementary table 2. Interview guide

Opening questions	Prompts
How long have you had asthma for?<br>Do you know the name of or can you describe the colour of your regular inhalers you use?<br>How long have you used those same ones for?<br>How do you feel about having asthma?<br>How do you feel about having to use inhalers every day?<br>What does asthma control mean to you?	How feel if didn't have inhalers?
**Understanding of own medication**	
Has a healthcare professional told you what medication is in your inhalers?	Have you been told they contain steroids?
Have you been told why your inhalers contain steroids?	Have you been informed that asthma is caused by inflammation in the airways and that steroids stop that inflammation?
Have you thought about if you will always need your inhalers, and if at these doses?	Has your asthma been the same for a long time?<br>Does anyone discuss long term prognosis and use of inhalers?<br>Have you asked a healthcare professional about long term plans with your asthma, if so, who?
How do you decide how often to use your inhalers?	Do you use an asthma management plan?<br>Do you use it for short- or long-term planning or both?
**Views on inhalers**	
What do you think about taking your asthma inhalers?	Are you happy taking your inhalers?<br>Are you happy with your inhaler technique? Has anyone shown you the correct technique or have you watched videos eg Asthma UK video?<br>When is the last time anyone checked it or asked you about it?
How often do you have to increase or decrease the amount you use them?	Do you think about taking them in the same way as if taking a daily tablet medication, or differently from other medication that you may take?<br>How many puffs a day do you use, does that change much eg during certain seasons or exercise-related or if have a cold?<br>Would you prefer to use your inhalers less often if your asthma remained stable?<br>How often have you had your inhaler changed to a different dose or inhaler?<br>Do you purposely try to reduce them, when (e.g. seasons, stable)?
**Knowledge on inhaler side effects and costs**	

Have you thought about if your inhalers have side-effects?<br><br>What do you think is the difference between steroid tablets and inhaled steroids?<br><br>Does the cost to *you* affect how you use your inhalers?<br><br>Does the cost to *the NHS* affect how you use your inhalers?	Have you asked about side-effects or spoken with anyone about that?<br>Are you aware of any steroid side effects? Can you tell me what they might be?<br><br>Do you have any conditions that might concern you if you are regularly using inhaled steroids?<br><br>Do you have osteoporosis, diabetes, eye problems, pneumonia, easy bruising, oral candida?<br><br>Do you pay for your prescriptions?<br>If so… are inhalers expensive for you?<br>How often do you get your inhalers?<br>If they were free would that change?<br><br>Do you think about the cost to the NHS?
**Experiences of stepping down by a professional**	
Has any healthcare professional discussed reducing your asthma medication with you?<br><br>If you did it, how did you feel about reducing it?<br><br>What happened after you reduced it?<br><br>If it was not successful, why do you think that was?<br><br>If you were asked to reduce it and you did not, why was that?	If yes… Who was that?; When was that?; Why did they?<br>Did you reduce it?; Did they explain why?<br>Did you trust the professional? Did you feel they knew what they were doing?<br>Did you get written or verbal instructions?<br>What were you worried about?<br><br>How much did you reduce by, which inhaler, how quickly?<br><br>Did they tell you what to do if your asthma worsened?<br>Was that written or verbal instructions?<br>Were you told to check your peak flow?<br>Did they plan to review you after?<br><br>What happened, did you stay on the lower dose or need to go back on a higher dose?<br><br>If you went back up again, how did that feel?<br>Would you try it again a different time?
**Views on stepping down in the future**	
If a healthcare professional advises you can reduce your inhaler dose, how would you feel?<br><br>Would you prefer to reduce the number of puffs or have a different inhaler, or not mind?<br><br>Have you ever read or been told that some inhalers are slightly better for the environment than others?<br><br>Why do you think a professional would ask you to reduce your inhaled medication dose?<br><br>Would having a plan of when and how to reduce it help? For example, like a management plan but for 6	Would you be happy to do that?<br>Why would you be happy?<br>Would you be worried about that and why?<br><br>Which would be easier for you?<br><br>Would you be interested in changing to a more environmentally friendly inhaler?<br><br>Do you think it would be to help you or to cut costs?<br>What would help you convince you that changing was for your benefit not to save the GP practice money?

to 12 months management with guidance for long-term management as well as short-term if your asthma worsens like the current management plan?	Could you manage it yourself, or prefer to be reviewed at your practice?<br>Would you measure your peak flow?

JM Reflexive account

I am one of the co-authors of this paper and an asthma sufferer. When I was first approached to be a part of this study, I had a special connection to it and hence was very keen to be a part of it. I have had asthma since I was a young child, I deem my asthma to be stable and well controlled. Now, working in healthcare, it has given me the opportunity to better understand my asthma in terms of my symptoms, triggers and the contents of my medications.

This paper had my engagement as soon as I met with my co-authors, especially when we discussed the stepping down of asthma medications. This is something that has very seldom been discussed with me from an asthmatic's perspective. I have been on the same medication and dose for at least four years with no formal follow up/annual review.

Throughout reading the transcripts, I could relate so closely to many participants regarding asthma care in general. Especially concerning the lack of education surrounding how to and when to step down medications and the lack of asthma reviews. As an asthmatic, I identified with the participants' attitude of wanting to step down their medications but wanting guidance on how to do this.

Recently, I have had a medication review with a clinical pharmacist, at this appointment I made a point of asking how I can step down my medications and spoke about my involvement in this paper. After the appointment it made me think how many other asthmatics ask questions like that and was I different because of my involvement in this paper and because I'm a healthcare professional. As a result of this appointment, I asked how I should step down my medications, in which I was advised to reduce the number of puffs in the morning and evening to half, i.e. one puff twice per day rather than two puffs twice per day. I asked if I should commence this immediately (March 2024) and I was advised to do this in the summer months. The pharmacist advised I book in with the practice asthma nurse for a review, sadly these appointments were in the middle of the working day. This made me

feel slightly frustrated by the fact not only have I not had a review in years, when I am offered one, it is made harder for a working individual to get a review.
